# A Model and Descriptive Case Example for Partnered Implementation Playbook Development

**DOI:** 10.1007/s43477-026-00233-6

**Published:** 2026-05-25

**Authors:** Sophia M. Bartels, Zenith Rai, Beth Ann Petrakis, Maura Dougherty, Vincent Song, Keith McInnes, Angela M. Kyrish, Deborah Lee, Antonio D. Harris, Jessica Blue-Howells, Bo Kim, Justeen K. Hyde

**Affiliations:** 1 VA Bedford Healthcare System, Bedford, USA; 2 VA Boston Healthcare System, Boston, USA; 3 Homeless Programs Office, Washington, USA; 4 Harvard Medical School, Boston, USA; 5 Boston University School of Medicine, Boston, USA

**Keywords:** Implementation playbook, Peer support, Post-incarceration, Implementation strategies

## Abstract

Implementation playbooks are guides for implementing evidence-based interventions and are tools for increasing their dissemination. However, there is little guidance on developing implementation playbooks in partnership with implementors, which is important for improving their relevance and usability. Our objective was to develop a model for partnered implementation playbook development and provide a descriptive case example for applying this model in practice. This work was conducted as part of an implementation study of a peer-based Post-Incarceration Engagement intervention. To address the dearth of guidance on implementation playbook development, our team documented the steps we took to create an implementation playbook for this intervention. We held iterative discussions to develop the model, which was further refined through feedback from our partners. The Implementation Playbook Development Model steps include: (1) Create playbook team and meet with partners to understand what they would like in a playbook; (2) Take stock of existing materials; (3) Develop and share preliminary playbook outline with partners for feedback; (4) Examine existing infrastructure for housing the playbook and determine appropriate format; (5) Assign playbook development tasks to playbook creation team; (6) Conduct internal review of the playbook; and (7) Share full playbook with partners for feedback and refinement. Creating our model through a partner-engaged process resulted in an online playbook for implementing the Post-Incarceration Engagement intervention that accounted for our partners’ needs, capacity, and infrastructure. This 7-step model and descriptive case example will be useful for others who are ready to disseminate effective interventions across healthcare settings and communities.

**Supplementary Information:**

The online version contains supplementary material available at 10.1007/s43477-026-00233-6.

While we know more about how to successfully implement evidence-based interventions (EBIs) through the growth of the global implementation science field, less is known about how to successfully disseminate and sustain them (Wiltsey Stirman et al., 2012). The result is that evidence-based interventions are not being effectively disseminated and less than half of EBIs are sustained long-term (Brownson et al., 2013; Wiltsey Stirman et al., 2012). Without knowledge about how to develop strategies that will support EBI dissemination and sustainment, the impact that EBIs can have will be greatly limited.

One strategy that may be effective for facilitating the implementation, spread, and sustainment of EBIs is implementation playbooks. Implementation playbooks provide step-by-step guidance on how to implement an EBI within a new organization or context and keep the implemented EBI going on a day-to-day basis. Thus, they can be referred back to even after initial implementation has taken place. This ideally enables conducting an EBI long-term without intensive support from the EBI developers (Brownson et al., 2013; Huynh et al., 2018). Implementation playbooks have been developed to support the spread of evidence-based digital mental health interventions, help community-based organizations plan for and assess behavioral health interventions, and select evidence-based healthcare services (Boustani et al., 2019; Mohr et al., 2025; Yakovchenko et al., 2022). Implementation playbooks are also considered to be important tools within Learning Health Systems, which are generally defined as healthcare organizations whose goals are to increase the speed at which EBIs are developed and translated into practice (A. M. Kilbourne et al. 2024a, b; Amy M. Kilbourne et al. 2024a, b).

Despite the potential of implementation playbooks, there is little guidance on how to develop them in practice. While previous research has identified methods, such as group model building and implementation mapping, for selecting and tailoring implementation strategies, these methods do not provide step-by-step instructions on how to create them from the ground-up (Powell et al., 2017). There are a number of published examples of implementation playbooks for specific interventions, like Getting to Implementation (Yakovchenko et al., 2022), and for broad approaches to implementation, like the WHO Implementation Playbook (Implementation playbook, delivering impact for health, 2023); however, while helpful examples, they do not provide step-by-step instructions for how to undertake the playbook development process.

Without detailed guidance, on a global scale, researchers may be less likely to undertake playbook development as an implementation strategy. Importantly, there is also little guidance for how to develop a playbook in partnership with key actors responsible for implementation, such as frontline staff, supervisors, and policymakers. Without partnership involvement, implementation playbooks will be less user friendly and accessible, factors that would diminish their use (Huang et al., 2018). While there is a growing literature around the use of established participatory and co-design methods to support the engagement of implementation partners in the design of implementation strategies (Archibald, 2023; Bernard et al., 2025; Tobiano et al., 2025), we lack concrete, step-by-step models for creating implementation playbooks in collaboration with implementation partners. Therefore, the objectives of this paper are to build on existing partnered-engaged methodologies to: (1) describe a model for partnered implementation playbook development and (2) provide a descriptive case example for applying this model in practice (Greenhalgh, 2025).

## Methods

### Model Development & Descriptive Case Example Context

Our partnered implementation playbook development model and descriptive case example emerged from work conducted within the context of the Veterans Health Administration (VHA) and with implementation partners within VHA’s Homeless Programs Office. Addressing and ending homelessness for Veterans has been a longstanding initiative for the VHA, spearheaded by the creation of a central Homeless Programs Office. The mission of the Homeless Programs Office is to assist Veterans and their families with obtaining permanent and sustainable housing and accessing high-quality healthcare and supportive services (*VA Homeless Programs, *2025). Our team’s work with the Homeless Programs Office was focused on improving support provided to Veterans leaving incarceration.

Veterans often face unique and significant challenges that may make post-incarceration reentry and reintegration into society difficult. Many Veterans have difficulty securing stable housing. They also face challenges such as navigating resource lists and bureaucratic processes required to apply for or re-obtain benefits, finding employment, and re-engaging in healthcare (Justeen Hyde et al. 2022a, b; Knudsen and Wingenfeld 2016; Richardson et al. 2025; Tsai and Rosenheck 2012). Without adequate support, the period after release from incarceration can be a risky time for Veterans. Prior research indicates that justice-involved Veterans are over three times more likely to have attempted suicide in their lifetime compared to non-justice involved Veterans (Holliday et al., 2021). The weeks immediately after release from prison tend to be the riskiest, as the rate of overdose and death are significantly elevated during this time, indicating the need for intervention (Hartung et al., 2023; Wortzel et al., 2012).

The VHA’s Homeless Programs Office established the Healthcare for Reentry Veterans program and the Veterans Justice Program in 2007 to address the needs of justice-involved Veterans (Finlay et al., 2017). The Healthcare for Reentry Veterans program uses social workers to provide outreach to incarcerated Veterans to support reentry planning. This reentry planning can include an assessment of needed treatment and other resources, identification of housing options, connection to health care treatment upon release, and assistance with other reentry needs (e.g., identification of employment opportunities and sources of social support). Although the program facilitates connections to VHA health care services, leaders within the Veterans Justice and Healthcare for Reentry Veterans programs realized that many Veterans needed greater assistance with the process of returning to community settings to support longer-term engagement in health and social services. Healthcare for Reentry Veterans programs faced time and resource constraints, limiting their provision of additional enhanced support during the critical post-release period that is important for Veterans’ longer-term stability (Hyde et al. 2022a, b).

### PIE Intervention and Scale-up

To address these challenges, our team developed the Post-Incarceration Engagement ​(PIE) intervention (Simmons et al., 2017). This intervention supplements the Healthcare for Reentry Veterans and Veterans Justice programs through the inclusion of a peer specialist (Veterans who have had similar life experiences, often incarceration, to the Veterans they served in the intervention) trained in the provision of PIE. PIE is grounded in evidence that peer specialists can help support engagement in healthcare and linkages to community supports (Hyde et al. 2022). Through PIE, trained peer specialists work to provide practical and emotional support to Veterans throughout the reentry process from pre-release to day-of release to post-release. Veterans Justice Program peer specialists provide four core types of support to Veterans: (1) social and emotional support, (2) linkage and referrals, (3) skill building and goal setting, and (4) community reintegration assistance (see Hyde et al. (2022) for more details about the PIE intervention). PIE has the overall goals of preventing Veteran homelessness and suicide, decreasing recidivism, and promoting Veterans’ connection to and engagement in VHA care. Prior evaluation studies found that the PIE intervention led to significant improvements in Veterans’ substance use and mental health treatment uptake and engagement, as well as higher rates of permanent housing and lower rates of recidivism among participants when compared to a historical comparison group (Hyde et al. 2022).

### Implementation Playbook Development Process

Given promising outcomes associated with the PIE intervention and its potential to be used by other VHA sites, our team sought to develop an implementation playbook for PIE (September 2024-August 2025). We undertook the playbook development process with our implementation partners from the Veterans Justice Program, leaders from the U.S. Department of Housing and Urban Development-Veterans Affairs Supportive Housing (HUD-VASH) Program, and Healthcare for Reentry Veterans program team members (described below). Our goal was to develop a digital playbook that the Veterans Justice Program could use as a tool and share with other VAs, ultimately encouraging PIE’s uptake across the country.

We sought models to support us in developing our implementation playbook. Yet, we found little guidance in the literature, and no guidance on how to develop implementation playbooks in partnership with implementors. Given this dearth of guidance, we decided to document the steps that our team took, in close collaboration with our implementation partners, to create the PIE playbook. The Partnered Implementation Playbook Development Model team included implementation scientists and anthropologists (with expertise in participatory methodologies), in addition to a project manager. The team used an adaptation of an iterative implementation science consensus approach (previously developed by members of our team using established decision-making methods) (Kalver et al., 2021) to create the Partnered Implementation Playbook Development Model. This process included brainstorming the steps our team used to create the PIE playbook, consolidating brainstormed steps into a draft model, sharing the draft model with our partners for feedback, incorporating suggestions from our partners into the draft model, and further refining the model through team discussions until there were no more suggestions to incorporate.

This work was funded by the VHA’s Health Services Research Quality Enhancement Research Initiative. The study was submitted to the Institutional Review Board at the VA Bedford Healthcare System (Bedford, Massachusetts, USA), which determined it was a quality improvement project as per Department of Veterans Affairs handbook 1200.05. The Standards for Quality Improvement Reporting Excellence guidelines were used as a framework for reporting this descriptive case example (Online Resource 1) (Ogrinc et al., 2008).

## Results

### Partnered Implementation Playbook Development Model (IPDM) & PIE Playbook Descriptive Case Example

**Fig. 1:**
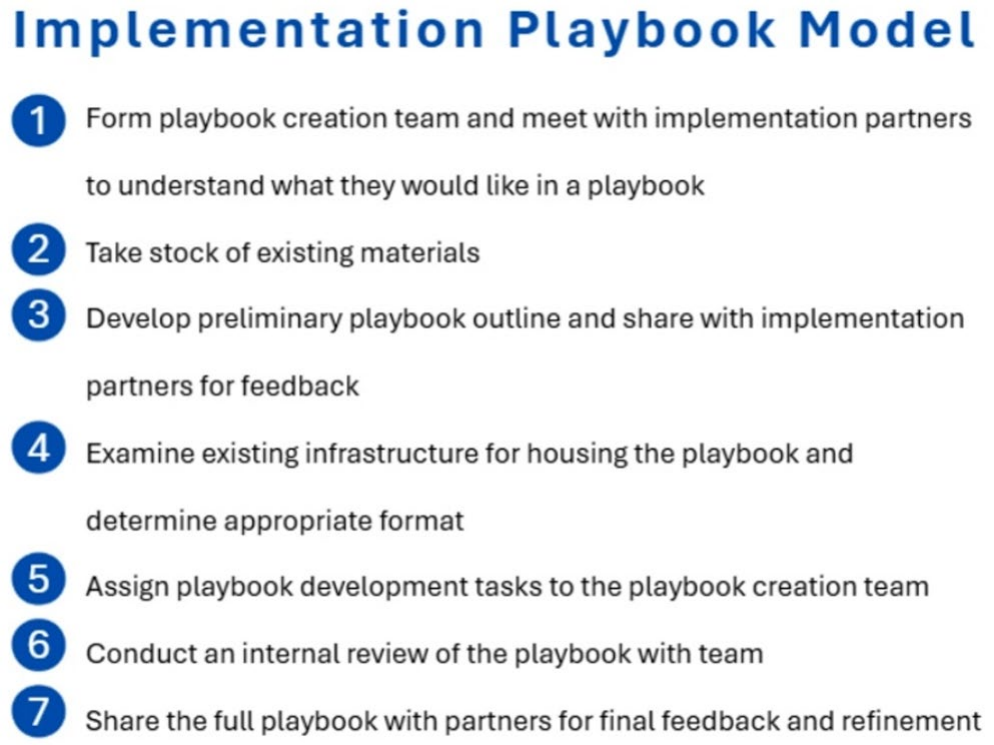
Implementation playbook development model steps

Our partnered Implementation Playbook Development Model (IPDM) includes seven steps: (1) form playbook development team and meet with implementation partners to understand what they would like in a playbook​; (2) take stock of existing materials; (3) develop preliminary playbook outline​ and share outline with implementation partners for feedback​; (4) examine existing infrastructure for housing the playbook and determine appropriate format​; (5) assign playbook development tasks to the playbook creation team​; (6) conduct an internal review of the playbook with the team​; and (7) share the full playbook with implementation partners for final feedback and refinement (Fig. 1). Below, we describe each step in detail as well as a descriptive case example of how we undertook it during the PIE project.

#### Step 1

The first step of the IPDM is to form a playbook creation team and meet with implementation partners to understand what they would like in a playbook. In forming a playbook creation team, we found that it was essential to include a combination of individuals with skills and expertise in (1) the intervention and its core components; (2) product design; (3) project management; (4) participatory methodologies; and (5) implementation science. This combination of skillsets ensured that the playbook contained all necessary intervention and implementation strategy-related elements and was visually appealing, as well as ensuring that the partnered playbook development process was well-organized and grounded in collaboration between the playbook creation team and implementation partners.

Once the playbook creation team is formed, meeting with implementation partners should be the first step of the playbook development process as it allows the team to center the playbook around partners’ needs from the beginning and will ensure that the team has accurate and informed knowledge of their needs. In thinking about who should be included in this process, it is important to incorporate partners from across implementation levels. We define implementation partners whose involvement is essential in the implementation playbook development process as 

(1) leaders who are responsible for facilitating implementation of the intervention and providing implementation support and (2) front-line intervention implementors. Including both types of implementation partners is important as they bring different knowledge and expertise to the implementation playbook development process. Implementation partners will likely have been identified and involved throughout the intervention development process, but established methods (such as stakeholder mapping and analysis) can be utilized to further ensure that all individuals responsible for larger-scale implementation of the intervention have been identified and engaged in playbook development (Franco-Trigo et al., 2020; Implementation playbook, delivering impact for health, 2023).

In the case of our playbook creation team (~ four core and four peripheral team members), we made sure to include individuals with content expertise in the PIE intervention, design and product development skills (our project manager), and a background in qualitative analysis, participatory methods, and implementation science. Our implementation partners included three Veterans Justice Program/HUD-VASH leaders and three Healthcare for Reentry Veterans team members (social workers, Veterans Justice Program peer specialists, and supervisors) who were involved in supporting or delivering the PIE intervention during our effectiveness and implementation trials. Veterans Justice Program/HUD-VASH leaders provided essential insight into their needs and capabilities related to the implementation of PIE that could be supported through an implementation playbook. Healthcare for Reentry Veterans team members shared specifics around what would be helpful for them to have in an implementation playbook to facilitate their direct delivery of PIE. These initial meetings with implementation partners were structured as listening sessions (Ingman et al., 2023), whereby a facilitator from our playbook creation team asked our implementation partners their perspectives on a series of questions (e.g., what tools would you most want to see in an implementation playbook?), but others could use different types of co-design methods (Slattery et al., 2020) to structure their meetings with implementation partners. The meetings took place over a few weeks and lasted ~ 1 hour per meeting.

#### Step 2

The second step is to take inventory of existing intervention and implementation materials​. While some of the implementation support materials that are included in an implementation playbook are unique to the intervention and implementation partners’ needs, they will likely include an introduction to the intervention (e.g., its value), support materials around intervention start-up at a new site (e.g., communicating with leadership about the intervention), intervention protocols/manuals, intervention training materials, intervention and implementation outcomes (e.g., RE-AIM, proctor’s taxonomy) (Glasgow et al., 1999; Proctor et al., 2011), and evidence of the intervention’s effectiveness. Often there are multiple versions of the same type of resource. Identifying the latest versions and putting them together in a “working playbook documents” folder is recommended for several reasons. First, it reduces confusion over which documents team members should be working on for the final playbook. Second, it provides a succinct way to assess the availability of existing materials and identify new ones that may have been recognized as important in Step 1.

In the case of PIE, there were many materials that had already been created as part of the project and/or by the Homeless Programs Office (e.g., training materials, internal guides) and in some cases existed in multiple places. Additionally, there were relevant resources from other VHA program office SharePoint sites, such as the Office of Mental Health, that we could point to as a resource for Peer Specialist guidelines. ZR looked through our online PIE project folders and placed the latest versions of the PIE materials in a designated “working playbook documents” folder. She also worked with our operational partners to identify resources that they had developed (e.g., peer supervision guidance) that were relevant to PIE to include in the playbook resources. Once this was done, she compiled a list of the available PIE resources to provide the team with a starting point for the playbook materials.

#### Step 3

Step three of the IPDM includes developing the preliminary playbook outline and sharing it with implementation partners for feedback. Before content development, getting feedback on a preliminary version of the playbook from partners is essential for incorporating their expectations and suggestions for the playbook’s components early on and identifying additional resources to support playbook implementation that need to be developed. Structured tools like implementation mapping may be utilized at this stage with implementation partners to support the identification of additional implementation strategies to overcome context-specific barriers to scale-up of the intervention (Fernandez et al., 2019).

It is important to hold multiple rounds of iterative feedback discussions with partners to ensure that the playbook directly meets their needs and is based on practical knowledge and their real-world capacity. These iterative feedback sessions can also build buy-in from partners, increasing the likelihood of the playbook’s long-term use, which should improve spread and sustainment. Working on the playbook outline with implementation partners also helps further identify and sort implementation support materials that will be included in the playbook into (1) the existing materials already in final form, (2) existing materials that needed to be updated/revised, and (3) materials that need to be created.

Our PIE playbook outline and associated implementation support materials changed significantly from our preliminary draft to our final, iterated version (see Online Resource 2). For example, based on suggestions from our Veterans Justice Program/HUD-VASH partners, we added an elevator pitch/executive summary. We did this because partners wanted a quick and engaging way to share the key elements of the PIE intervention and why it was important, as well as a “pitch” for clinic leadership on PIE. Additionally, based on discussions with Veterans Justice Program peer specialists, we decided to create Veteran case examples, with a strong focus on combatting stigma (described below), to support peer specialists in working with more challenging cases.

These conversations also resulted in changes to pre-existing playbook materials. The Veterans Justice Program peer specialists shared that although the digital PIE intervention manual had useful information in the appendices, such as checklists and information sheets, they never used them due to the length of the manual. Because of this, our team decided to divide the manual into shorter, printable portions so that Veterans Justice Program peer specialists could bring the applicable portion(s) with them when they conducted their reentry work in the community.

We also made significant revisions to the outcomes and evaluation section of the playbook. Our original outcomes section focused on implementation outcomes, like reach and fidelity (Glasgow et al., 1999; Proctor et al., 2011). However, our Veterans Justice Program/HUD-VASH partners shared that the outcomes they were most interested in and resourced to measure were process outcomes. Examples of these metrics included: “are Veterans who have not previously had access to VHA being connected to services” and “does the Veterans Justice Program peer specialist have access to facilities needed to do Veteran outreach.” Finally, through these conversations the team discussed several additional existing resources that we had to revise and/or to which we could provide a link.

#### Step 4

The fourth step is to examine existing infrastructure for housing the playbook and determine the appropriate location and format​ (e.g., paper, online) for it. For long-term use of the playbook, it is essential to work with implementation partners to identify a platform that matches their capability and needs and will be easy for new implementors, or those considering implementing the new intervention, to use. For example, if partners have little bandwidth for platform management, it will likely be important to select a platform and location for the playbook that they are already using, will not require substantial ongoing maintenance, and is a “central hub” for sharing information.

In the case of the PIE playbook, while we had initially brainstormed some creative ways to house the playbook materials, our partners shared that they would have limited capacity to manage anything more complicated than the online SharePoint site that they currently use for their program materials. With this approach, user access to the sites would not be challenging. Because other resources already existed there, it made sense to house the playbook on their SharePoint site and for the playbook to use the same structure as their currently existing resources (i.e., subfolders within folders). In considering the longevity of the online playbook, we also discussed the use of hyperlinks to outside resources. Because outside links to online resources and websites tend to break over time, we only included in the playbook links to resources that were already located within the SharePoint site instead of to external links. Future users of the IPDM could also link to materials stored in shared drives.

#### Step 5 & 6

Steps five and six include assigning playbook development tasks to the playbook creation team and conducting an internal review of the playbook. Assigning playbook development tasks among the team ensures that tasks are divided based on expertise and knowledge (e.g., content writing, design, visual products). For example, the project leads who developed and pilot tested the PIE intervention were assigned the task of reviewing and updating the content of the PIE intervention manual. One teammate who was gifted in visual design developed an introductory video providing a brief overview of the PIE intervention (described below) and revised materials to make them visually appealing and engaging. Another team member who had expertise in qualitative analysis developed case examples from Veteran interview transcripts.

Once the materials have been drafted, an internal review is conducted among the playbook creation team of language clarity, material accuracy, and usability. This internal review ensures that the playbook is aligned with partners’ goals and is appropriate and accessible to them.

#### Step 7

The last step in the IPDM involves sharing the full playbook with partners for final feedback and refinement​. While this step is listed as the last, in reality, steps 5–7 occur iteratively, with the team developing content, reviewing it internally, sharing it with partners, and revising it based on their feedback. This ensures that final adjustments and feedback from partners are incorporated into the playbook and that no components are finalized without their thorough review. In the case of the PIE playbook, this partner-engaged process ensured that the playbook accounted for their needs and aligned with their capacity and infrastructure, increasing the likelihood of its long-term use.

### Example PIE Playbook Products

Examples of products developed and/or updated as part of the IPDM for the PIE project included (1) a brief video introduction to the PIE intervention, (2) implementation roadmap, (3) PIE training manual, (4) PIE training videos, (5) Veteran case examples, and (6) a resource folder to support peer specialists’ work with Veterans (e.g., technology guidance for Veterans, example daily planner, resources to facilitate conversations about what matters most to Veterans, brochures, etc.). As mentioned above, we created the *introduction to PIE video* to be an engaging product geared towards hospitals and medical centers that did not have prior knowledge of PIE (*email authors for access to video*). Our goal was that Veterans Justice Program/HUD-VASH leaders would be able to share this video with VHA leaders around the country to build interest in using the PIE intervention. Similarly, the goal of the *implementation roadmap* was to provide clinic leadership, who were considering adopting PIE, with a high-level, practical overview on what it would actually take to implement it (see Online Resource 3 for roadmap). The PIE video and implementation roadmap were developed for the playbook directly based on our partners’ ideas and needs.

The next three materials, the PIE training manual, recorded training videos, and Veteran case examples were developed and/or modified to be used by facilities that were starting to or were already implementing PIE. The *PIE training manual* was a central tool that had already been developed. Necessary revisions to the manual discussed during IPDM steps 3 and 4 included removing details about the implementation support that was originally provided by the intervention team, updating language throughout the manual to be inclusive of Veterans returning to community settings from both prisons and jails (original focus of the PIE intervention was on prisons), and adding information about legal needs and medical-legal services available through VHA to assist with meeting them.

As mentioned above, a new material that we developed based on feedback from the Veterans Justice Program peer specialists was the *Veteran case examples*. The Veterans Justice Program peer specialists shared that it would be helpful for them to have examples of typical and challenging cases, particularly related to incarceration stigma, that they could use to help guide their work with Veterans. From this feedback, the team aggregated and anonymized interview data from Veterans who had gone through the PIE program to create three case examples (with significant input from our partners): (1) John- an older Veteran with little social support and complex medical conditions, (2) Danny- a 45-year-old Veteran with a previous substance use disorder, and (3) Rick- a 30-year-old Veteran with a sex offense and significant family support (see Online Resource 4 for Veteran case examples).

In addition, the *technology guidance for Veterans* resource was developed based on conversations with peer specialists and some of the reentry Veterans working with them. Veterans who had been incarcerated for many years were particularly disadvantaged when they returned to community settings given their lack of experience with or understanding of many of the technologies used in everyday life (e.g., ATM machines, self-check-out kiosks, smartphones, etc.). The *technology guidance for Veterans* document was developed as a resource to include in the playbook.

## Discussion

As part of our evaluation of the implementation of the PIE intervention within the VHA, we developed a partnered Implementation Playbook Development Model. The IPDM includes seven steps that researchers and implementors can follow to create implementation playbooks for their EBIs, in close collaboration with their implementation partners.​ These steps span from meeting with partners to understand what they would like in a playbook to sharing the full playbook with partners for final feedback and refinement. One of the main strengths of this work is that, as far as we know, this is the first model for developing implementation playbooks, particularly with a focus on collaboration with partners.

We also provided a descriptive case example of applying the IPDM to develop a playbook for the PIE intervention with our partners in the VHA Homeless Programs Office. In this descriptive case example, we demonstrated how following the 7-step IPDM led to the creation of a highly usable and accessible playbook that fully accounted for our partners’ needs and capacity. As part of this descriptive case example, we provided examples of the implementation playbook materials that we developed or updated with our partners, such as an introduction to PIE video, implementation roadmap, and Veteran case examples. Researchers and implementors will be able to use this descriptive case example, and associated playbook materials, to inform and support their use of the IPDM to develop playbooks with their implementation partners.

The central focus of our playbook development process was to build off previously existing methods for partner engagement (Slattery et al., 2020) to address the lack of step-by-step, practical guidance on *what to do* to develop an implementation playbook in collaboration with partners and *how to do it*. Throughout our playbook development process, we experienced the immense value of this partnership. This finding is in alignment with previous work identifying partnership as the “backbone of implementation science” (Beidas et al., 2022). We found that this partnership increased our partners’ perceptions of the implementation playbook’s utility, helped us streamline our existing materials to avoid the use of old or outdated resources, and supported us in creating additional materials that may encourage greater spread of the playbook to other VHA facilities. For example, through our partnership, we developed the PIE video and elevator pitch, roadmap, and Veteran case examples. Those products have the potential to encourage PIE’s dissemination and adoption, from awareness raising about the intervention, to facilitating contemplation about its applicability to a VHA facility, to increasing its effectiveness, respectively.

This intensive level of partnership may be novel to some researchers and operations staff. If this is the case, start-up time will need to be allotted for them to develop their collaboration style and select methods for partnership. As part of this preparation phase, researchers should determine processes they will undertake to manage power dynamics (both between the playbook creation team and implementation partners and amongst the partners themselves) and to navigate conflicting opinions and recommendations that may arise from partners during the playbook creation processes. The co-design field has developed processes for addressing these challenges that can be utilized throughout the IPDM, such as through intentional establishment of trustworthiness and norms for managing conflict (Israel, 2013).

We believe that the use of this partnership-centered model may help ensure that playbooks are truly relevant to partners and help increase the likelihood that the work of developing the EBI will not “end with publication” (Chambers & Azrin, 2013). The IPDM was developed so that all steps encourage sustained use of the playbook over time. For example, in designing the playbook with our partners, we made sure to select a platform that would enable long-term use of and access to the playbook. Additionally, in choosing implementation partners for the playbook’s design, we included individuals from across the implementation spectrum (e.g., from Veterans Justice and HUD-VASH program leadership to Veterans Justice Program peers and social workers using the PIE model) to increase multi-level buy-in and ensure acceptability and utility of the playbook from all parties who would be involved with PIE’s implementation. By designing the IPDM with a focus on dissemination and sustained use from the start, we hope to support greater and longer-term use of implementation playbooks, with the ultimate goal of increased uptake of evidence-based interventions across healthcare systems and communities (Kwan et al., 2022).

Another lesson from this process is that when creating a playbook, it is essential to allocate sufficient time and effort into the playbook’s visual design to create engaging products that will be simple for implementation partners to use and encourage uptake. Grounding playbook materials in strong visual design is aligned with the dissemination literature that describes how design elements, such as simplicity, organization, and content utility, can help increase user engagement (Garett et al., 2016; Lord et al., 2019).​ While having a strong visual design makes a playbook more aesthetically pleasing, it can also increase functionality. A well-designed playbook can play a critical role in how easily end users can navigate and understand the materials. Focusing on visual design and incorporating cleaner formats, visual cues, and visual aids can help organize information clearly. In our playbook, we considered visual design intentionally throughout the development process, not just in the final stages. We spoke with end users (peer specialists) to identify which visual formats were most useful and likely to result in consistent use (e.g., breaking the manual into shorter, printable sections that they could bring with them into the community). Investing in thoughtful visual design helped ensure that implementors and end users would not only look at the materials, but also consistently turn to and use them as a guide.

One limitation of this work is that while it emerged directly from our experience developing an implementation playbook with our partners for the PIE project, this model has yet to be used directly to support implementation of other interventions, which may reduce its transferability. However, the IPDM was shared with two additional implementation studies that were funded under the same Quality Enhancement Research Initiative Center. Leads of these studies utilized the playbook guidance to inform their approach to developing their own playbooks. They reported utility of the guidance, which supported our decision to share it more broadly. Nevertheless, more direct evaluation of the IPDM is needed before conclusions can be made about its impact on improving implementation and spread of EBIs.

A second limitation of the IPDM is that it was developed within the context of the U.S. Veterans Health Administration and has yet to be tested in countries outside of the U.S. While the seven steps included in the IPDM are purposefully broad enough that they can be transferable to development of playbooks in other settings, some of the details within the steps may need to be fine-tuned or adapted to fit global contexts in which they are being implemented (e.g., considerations for playbook storage and dissemination in contexts with limited internet access, societal hierarchies that may necessitate additional considerations around implementation partner selection and engagement) (Jagtap, 2022).

## Conclusions

Little guidance exists for how to create an implementation playbook collaboratively with implementation partners. This partnered Implementation Playbook Development Model and associated case example provides steps that researchers and practitioners can undertake to develop an implementation playbook, when they are ready to disseminate an effective intervention more broadly.

## Supplementary Information

Below is the link to the electronic supplementary material.

Supplementary Material 1

Supplementary Material 2

Supplementary Material 3

Supplementary Material 4

## Data Availability

Data sharing is not applicable to this article as no datasets were generated or analysed during the current study.
