# Supplementary material for: A Model and Descriptive Case Example for Partnered Implementation Playbook Development
Source: Glob Implement Res Appl. 2026 May 25;6(3):426–35. doi: 10.1007/s43477-026-00233-6 (PMC13481762; doi:10.1007/s43477-026-00233-6)
Supplement: Supplementary file 2 [file 43477_2026_233_MOESM2_ESM.docx]

**Online Resource 2**

**Article Title**: A model and descriptive case example for partnered implementation playbook development

**Journal Name**: *Global Implementation Research Applications*

**Author Names and Affiliations**: Sophia M. Bartels^1^, Zenith Rai^1^, Beth Ann Petrakis^1^, Maura Dougherty^2^, Vincent Song^1^, Keith McInnes^1^, Angela M. Kyrish^1^, Deborah Lee^3^, Antonio D. Harris^4^, Jessica Blue-Howells^4^, Bo Kim^2,5^, Justeen K. Hyde^1,6^

^1^US Department of Veteran Affairs, Center for Health Optimization and Implementation Research, VA Bedford Healthcare System

^2^US Department of Veteran Affairs, Center for Health Optimization and Implementation Research, VA Boston Healthcare System

^3^US Department of Veteran Affairs, Homeless Programs Office

^4^US Department of Veteran Affairs, Veterans Justice Program, Homeless Programs Office

^5^Department of Psychiatry, Harvard Medical School, Boston, MA, USA

^6^Department of General Internal Medicine, Boston University School of Medicine, Boston

**Correspondence to**: [sophia.bartels@va.gov](mailto:sophia.bartels@va.gov)

PIE Playbook Preliminary vs. Final Draft
